# Expanding the adenovirus toolbox: reporter viruses for studying the dynamics of human adenovirus replication

**DOI:** 10.1128/jvi.00207-24

**Published:** 2024-04-19

**Authors:** Cason R. King, Mackenzie J. Dodge, Katelyn M. MacNeil, Tanner M. Tessier, Joe S. Mymryk, Andrew Mehle

**Affiliations:** 1 Department of Medical Microbiology and Immunology, University of Wisconsin-Madison, Madison, Wisconsin, USA; 2 Department of Microbiology and Immunology, University of Western Ontario, London, Ontario, Canada; 3 Division of Protective Immunity, The Children’s Hospital of Philadelphia, Philadelphia, Pennsylvania, USA; 4 Department of Pathology and Laboratory Medicine, University of Pennsylvania Perelman School of Medicine, Philadelphia, Pennsylvania, USA; 5 Department of Oncology, University of Western Ontario, London, Ontario, Canada; 6 Department of Otolaryngology, University of Western Ontario, London, Ontario, Canada; 7 London Regional Cancer Program, Lawson Health Research Institute, London, Ontario, Canada; International Centre for Genetic Engineering and Biotechnology, Trieste, Italy

**Keywords:** reporter virus, fluorescence, bioluminescence, NanoLuc, adenovirus, plaque assay, viral kinetics, gene expression, viral replication compartments, microneutralization assay

## Abstract

To streamline standard virological assays, we developed a suite of nine fluorescent or bioluminescent replication competent human species C5 adenovirus reporter viruses that mimic their parental wild-type counterpart. These reporter viruses provide a rapid and quantitative readout of various aspects of viral infection and replication based on EGFP, mCherry, or NanoLuc measurement. Moreover, they permit real-time non-invasive measures of viral load, replication dynamics, and infection kinetics over the entire course of infection, allowing measurements that were not previously possible. This suite of replication competent reporter viruses increases the ease, speed, and adaptability of standard assays and has the potential to accelerate multiple areas of human adenovirus research.

**IMPORTANCE:**

In this work, we developed a versatile toolbox of nine HAdV-C5 reporter viruses and validated their functions in cell culture. These reporter viruses provide a rapid and quantitative readout of various aspects of viral infection and replication based on EGFP, mCherry, or NanoLuc measurement. The utility of these reporter viruses could also be extended for use in 3D cell culture, organoids, live cell imaging, or animal models, and provides a conceptual framework for the development of new reporter viruses representing other clinically relevant HAdV species.

## INTRODUCTION

As obligate intracellular parasites, all viruses are critically dependent upon the host cell. Intensive selective pressure, rapid replicative cycle times, and severe restrictions on viral genome size combine to drive virus evolution. As a consequence, viruses have been forged into exquisitely sophisticated instruments that functionally reprogram the infected cell (1–4).

Human adenoviruses (HAdV) are endemic human pathogens that cause conjunctivitis, gastroenteritis, hepatitis, myocarditis, and pneumonia (5). Although HAdV outbreaks mainly cause acute respiratory disease, severe illness is more commonly observed in immunocompromised individuals, where disseminated infection can be fatal (5).

Studies of HAdV have a long history of teaching us about cell biology. For example, HAdV was the first human virus shown to be oncogenic (6) and mRNA splicing was originally independently described by Dr. Phillip Sharp and Dr. Richard Roberts, resulting in a Nobel prize based on their work with HAdV (7, 8). Studies of this small DNA tumor virus have repeatedly illustrated the profound impact of viral proteins on multiple host functions to maximize viral propagation (9–15), providing a wealth of knowledge regarding oncogenesis, cell cycle control, DNA replication, transcription, mRNA processing, immunological response, and apoptosis. In addition, the well-studied and readily manipulated HAdV genome is the foundation for vectors for gene therapy and oncolytic viruses (16, 17). Furthermore, adenoviruses have also demonstrated great utility as a vaccine platform, with a recent example including the Oxford-AstraZeneca modified chimpanzee AdV vector vaccine for severe acute respiratory syndrome-coronavirus 2 (SARS-CoV-2) (18, 19).

While adenoviruses have been thoroughly studied, there is still untapped potential to be discovered as we further our understanding of these viruses through more advanced research techniques. In particular, reporter viruses constructed to provide simple quantitative readout are routinely used to accelerate studies of other viruses. While many previous HAdV reporter viruses have been generated, these have often been on an as-needed basis to address a specific research purpose. As such, they were not initially designed with broad utility. Indeed, these reporter viruses often lack the E3 immune evasion region or were engineered in specific oncolytic platforms containing additional genetic alterations (20–26). Beginning with a fully wild-type (WT) HAdV-C5 genome, we have created a versatile panel of nine fluorescent or bioluminescent reporter viruses that replicate similarly to the parental virus in culture, express reporters with early or late gene kinetics, and display robust fluorescence or bioluminescence. These reporter viruses enable rapid analysis of viral gene expression and the dynamics of the adenovirus replication cycle, which will accelerate multiple areas of HAdV research.

## RESULTS

### Design and growth kinetics of reporter adenoviruses

**Fig 1:**
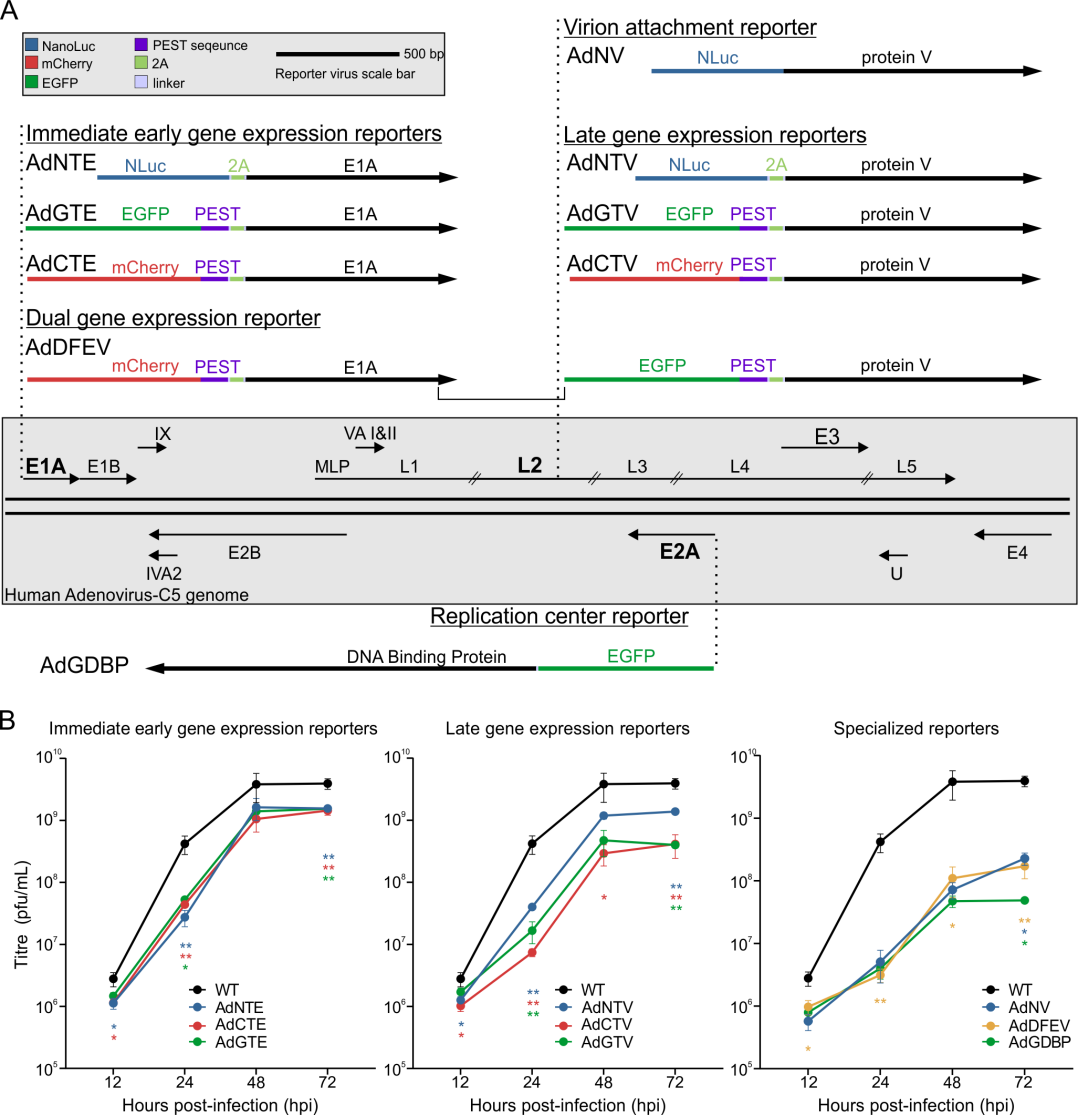
The reporter adenoviruses mirror WT growth kinetics. (**A**) A visual representation of the nine reporter viruses engineered into the HAdV-C5 genome. A legend and scale bar are shown in the top left corner. AdNTE, AdGTE, and AdCTE were used to report on early viral gene expression, whereas AdNTV, AdGTV, and AdCTV report on late viral gene expression. These reporter elements are connected through a 2A sequence to E1A or pV. AdDFEV is a dual fluor mCherry-2A-E1A and EGFP-2A-V virus. The reporter elements of the fluorescent viruses are followed by a PEST sequence for improved temporal resolution. AdNV creates bioluminescent virions to study viral attachment. Finally, AdGDBP has EGFP fused to the E2A-encoded DBP to study viral replication centers. All viruses were constructed using the AdenoBuilder platform. (**B**) Viral growth kinetics of the immediate early gene expression reporter viruses, late gene expression reporter viruses, and specialized reporter viruses are shown with the same WT data in each graph for ease of comparison. A549 cells were infected with the indicated reporter virus for 12, 24, 48, or 72 h before using supernatant to measure viral yield via plaque assay. Error bars represent standard error of the mean*<0.05, **<0.01, ***<0.001 (*n* = 3).

Using the AdenoBuilder platform, we constructed nine reporter viruses, in addition to a WT control, to investigate HAdV-C5 replication kinetics (27). Reporter viruses were created using either EGFP or mCherry, as they are commonly used fluors with widespread equipment availability, or NanoLuc (NLuc) as a small and sensitive enzymatic reporter. Immediate early gene and late gene expression reporters were constructed by adding either NLuc, EGFP, or mCherry, upstream of the viral E1A (Fig. 1A: AdNTE, AdGTE, and AdCTE) or Protein V (pV) gene (Fig. 1A: AdNTV, AdGTV, and AdCTV), respectively. Reporters were linked to viral proteins with a porcine teschovirus 2A sequence, which self-cleaves upon translation to uncouple reporter activity from the protein of interest to reduce attenuation of viral protein function (28, 29). These reporters also contain a murine ornithine decarboxylase proline – glutamic acid – serine – threonine (PEST) degron sequence downstream of the respective reporter genes, to enhance protein turnover and more accurately capture expression dynamics (30). A dual fluor E1A and pV reporter virus (Fig. 1A: AdDFEV) was created by combining the elements of AdCTE and AdGTV to make a virus expressing distinct reporters in the early and late stages of infection. A virion attachment reporter virus (Fig. 1A: AdNV) was constructed with an upstream NLuc fused to the pV gene, without a 2A cleavage site, ensuring NLuc is incorporated into virions, making them bioluminescent. Finally, the replication center reporter (Fig. 1A: AdGDBP) consists of the EGFP fluor fused to the E2A-encoded DNA binding protein (DBP).

Recombinant viruses were rescued, plaque purified, amplified, and confirmed by sequencing. These viruses were used to initiate multicycle infections in human A549 lung cells. Viral titers were measured by plaque assay at 12, 24, 48, and 72 hpi to compare their viral growth kinetics to that of WT HAdV-C5 (Fig. 1B). Each single gene reporter virus with a 2A sequence showed a lower viral yield than the WT at all measured timepoints. The uncleaved fusion reporters and the dual expression reporter exhibited a further reduction in viral yield than the single gene reporters in comparison to WT. Despite these growth deficits, the overall growth kinetics of all reporter viruses resembled the same pattern shown by WT HAdV-C5, with a sharp increase in viral yield from 12 to 48 hpi, followed by a plateau between 48 and 72 hpi (Fig. 1B).

### Immediate early viral gene expression reporters faithfully recapitulate HAdV-C5 early phase kinetics

**Fig 2:**
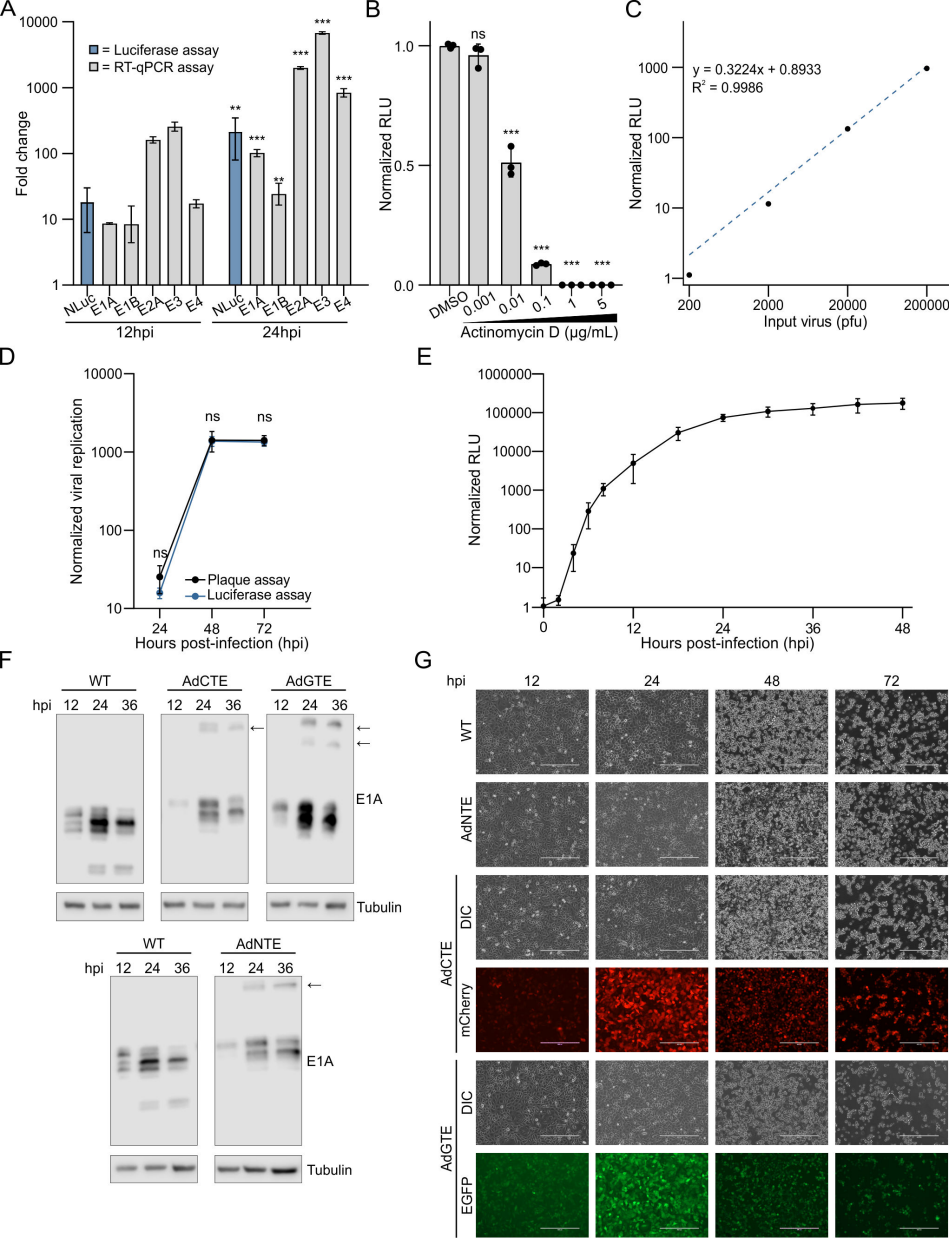
Immediate early viral gene expression reporters accurately capture HAdV-C5 early phase kinetics. (**A**) Comparison of RT-qPCR for early adenovirus gene expression with reporter luciferase activity for the AdNTE reporter virus. A549 cells were infected with AdNTE for 12 or 24 h before mRNA was harvested for RT-qPCR analysis of the early adenovirus genes E1A, E1B, E2A, E3, and E4 (grey bars). Luciferase activity (blue bars) was also calculated at 12 and 24 h postinfection. Data are normalized to 6 hpi. (**B**) AdNTE RLU production is reliant on RNAPII activity. A549 cells were infected with AdNTE and incubated with increasing amounts of ActD to inhibit RNAPII activity. Data are normalized to the DMSO control. (**C**) AdNTE MOI is linearly related to RLU output across 4-log units. RLU values are normalized to 200 pfu input virus. (**D**) Luciferase activity and plaque titer of the AdNTE reporter virus showed a near-identical increase when normalized to 12 hpi. A549 cells infected with AdNTE for 24, 48, and 72 h, supernatants were harvested, and virus was titrated by luciferase assay (blue) and plaque assay (black). Data are normalized to titers at 12 hpi. (**E**) Time course of viral gene expression in A549 cells infected with AdNTE virus as measured by luciferase activity. Data are normalized to 0 hpi. (**F**) Western blots for E1A in cells infected with AdNTE, AdCTE, and AdGTE reporter viruses. The large E1A isoforms were detected using mouse anti-E1A (clone M73). Arrows indicate residual uncleaved reporter-E1A proteins. Tubulin was used as a loading control. (**G**) Microscopy of cells infected with AdNTE, AdCTE, and AdGTE reporter viruses for 12, 24, 48, and 72 h. Fluorescent detection of infected cells is shown by mCherry or EGFP, indicating active early viral gene expression. Data are shown as the grand mean of three biological replicates ± standard error of the mean. Significance was tested with (**A, D**) a Student’s *t*-test or (**B**) an ANOVA with Dunnett correction (*<0.05, **<0.01, ***<0.001). Scale bars represent 400 µm.

To determine the utility of our immediate early viral gene expression reporters, we verified that these reporters function as intended, and that this comes at minimal cost to viral fitness and protein activity. AdNTE (Adenovirus NLuc-2A-E1A) was designed to quantify changes in early viral gene expression using a rapid, simple, and quantitative luciferase assay. Indeed, cells infected with AdNTE displayed a log unit increase in RLU production at 24 hpi compared to 12 hpi, mirroring the increase in each early viral transcript detected through standard RT-qPCR analysis (Fig. 2A). Additionally, AdNTE-infected cells treated with increasing concentrations of the RNA polymerase II (RNAPII) transcription inhibitor, actinomycin D (ActD), induced a dose-dependent decrease in RLU production, demonstrating that AdNTE reporter activity is dependent on transcription from incoming viral genomes (Fig. 2B).

Early viral gene expression is proportional to the number of incoming viral genomes during infection (31). To test this with AdNTE, cells were infected with increasing amounts of the reporter virus, and bioluminescent signal was measured 6 hpi. We detected a linear relationship across 4 log units between normalized RLU production and input virus, indicating that AdNTE accurately reported changes in RLU that reflected the differences in quantities of incoming viral genomes (Fig. 2C). To test whether this relationship could be exploited to measure viral titers and identify changes in viral progeny production, A549 cells were infected with AdNTE, and viral supernatants were harvested at various timepoints postinfection. Supernatants were titrated using classic plaque assays or luciferase assays. The growth curves from both assays were effectively superimposable at all time points (Fig. 2D). This demonstrates bioluminescent quantification of infectious viral progeny using AdNTE is as accurate as quantification by standard plaque assay (Fig. 2D). Moreover, titer determined by luciferase assay could be performed only 6 h after infection, as opposed to plaque assays that require 7 days or more before measurements are complete. We performed a time course of AdNTE infections measuring luciferase expression over the 48 h infection. Bioluminescence increased rapidly from 2 to 18 hpi, followed by a plateauing signal that stabilized from 24 to 48 hpi (Fig. 2E). Cells infected with AdNTE showed a similar pattern of increasing E1A protein expression from 12 to 36 hpi and similar cytopathic effect compared to WT HAdV-C5 infections at these time points (Fig. 2F and G).

We created fluorescent E1A reporter viruses to enable visual, real-time measures of viral gene expression. For both fluorescent AdGTE and AdCTE (Adenovirus EGFP-2A-E1A and Adenovirus mCherry-2A-E1A) early gene reporters, infected cell fluorescence peaked at 24 hpi and tapered off at 48 and 72 hpi (Fig. 2G), mimicking that of E1A expression through the HAdV replication cycle (32, 33). Furthermore, E1A protein expression for these reporter viruses at early timepoints during infection (12–36 hpi) was similar to WT E1A expression (Fig. 2F). Thus, we have developed both bioluminescent and fluorescent reporter viruses that accurately reflected WT early gene expression and allowed for rapid, quantitative measures of viral gene expression and titers.

### Late viral gene expression reporters reproduce the HAdV-C5 late phase kinetics

**Fig 3:**
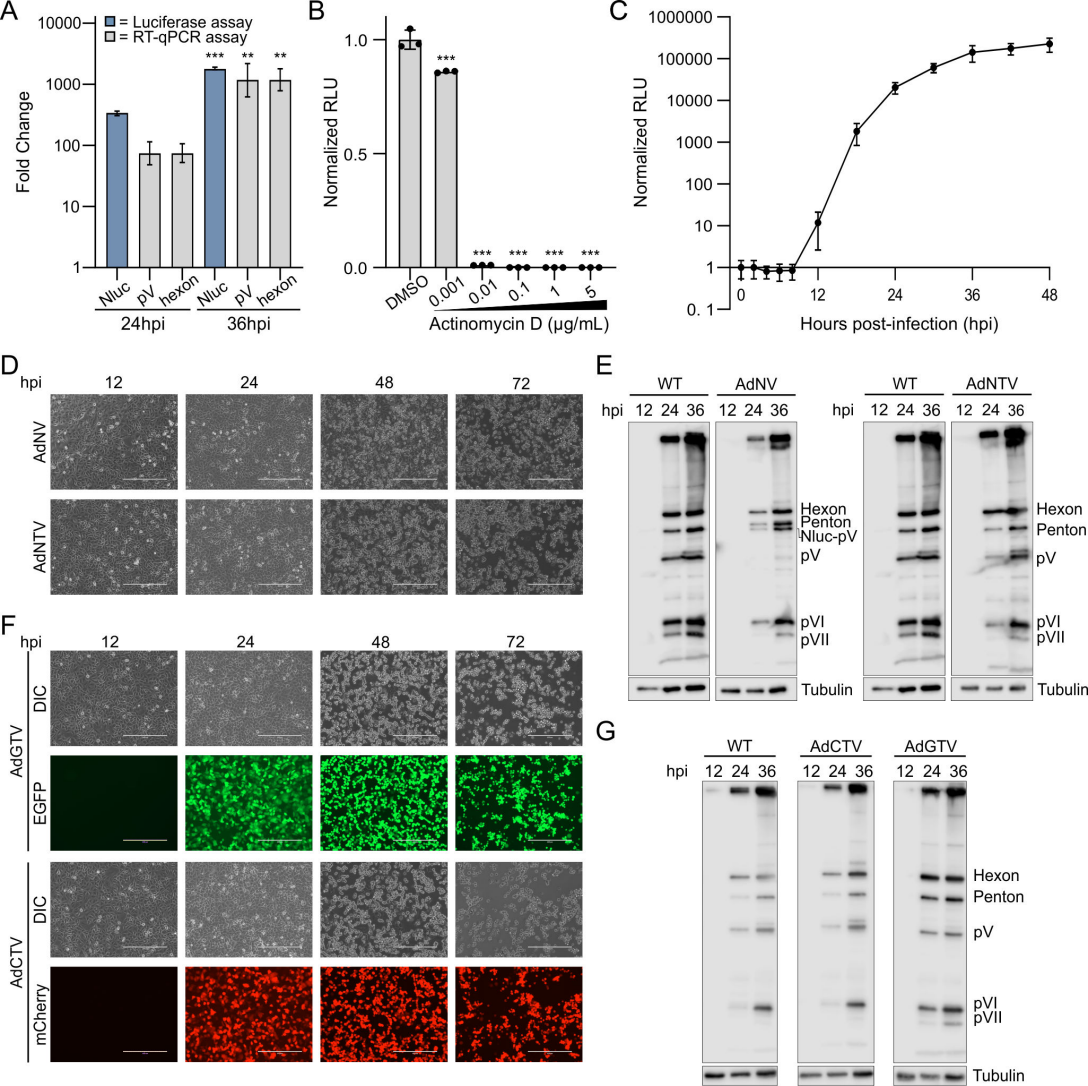
Late viral gene expression reporters reflect late phase HAdV-C5 kinetics. (**A**) Comparison of reporter luciferase activity and expression of the late viral genes encoding pV and hexon via RT-qPCR for the AdNTV reporter. A549 cells were infected with AdNTV for 24 or 36 h before infected-cell mRNA was harvested and subject to RT-qPCR analysis for the late adenovirus hexon- and pV-encoding genes (grey bar). Luciferase activity (blue bars) was also calculated at the 24 and 36 hours postinfection. Data are normalized to 12 hpi. (**B**) AdNTV RLU production is reliant on RNAPII activity. A549 cells were infected with AdNTV and incubated with increasing amounts of ActD before measuring late gene expression with a luciferase assay. Data are normalized to the DMSO control. (**C**) Time course of late viral gene expression in A549 cells infected with AdNTV reporter virus. Data are normalized to 0 hpi. (**D**) Microscopy of cells infected with AdNTV or AdNV fusion reporter viruses for 12, 24, 48, and 72 h. Cells infected in the same experiment with WT virus are shown in Fig. 2G. (**E**) Anti-Ad5 western blots of cells infected with AdNV fusion or AdNTV reporter viruses compared to that of WT HAdV-C5. Tubulin was used as a loading control. The same WT blot is shown twice for easy comparison to different reporter viruses. (**F**) Microscopy of cells infected with AdGTV or AdCTV reporter viruses for 12, 24, 48, and 72 h. Fluorescent detection of infected cells is shown by mCherry or EGFP, indicating active late viral gene expression. (**G**) Anti-Ad5 western blots of cells infected with AdCTV or AdGTV reporter viruses, compared to that of WT HAdV-C5. Tubulin was used as a loading control. Data are shown as the grand mean of three biological replicates ± standard error of the mean. Significance was tested with (**A**) Student’s *t*-test or (**B**) an ANOVA with Dunnett’s correction (*<0.05, **<0.01, ***<0.001). Scale bars represent 400 µm.

We tested our late viral gene expression reporter viruses to determine their utility in measuring changes in late viral phase kinetics. AdNTV (Adenovirus NLuc-2A-V) uses luciferase activity to report on changes to pV expression, a prototypical late gene product. We detected a significant increase in RLU production in AdNTV-infected cells at 36 hpi compared to 24 hpi, mirroring the change in gene expression of pV and hexon as measured by RT-qPCR (Fig. 3A). Similar to AdNTE, cells infected with AdNTV exhibited a decrease in RLU production upon treatment with increasing concentrations of ActD (Fig. 3B). A detailed time course of AdNTV infection revealed the expected increase of RLU production, with low levels of bioluminescence detected 10 hpi followed by a continuous increase in signal from 18 to 48 hpi (Fig. 3C). Luciferase expression was delayed during AdNTV infection versus AdNTE (Fig. 2E and 3C), reflecting its expression from the major late promoter.

Analysis of cell morphology over time revealed a similar cytopathic effect in cells infected with either AdNTV or the AdNV (Adenovirus NLuc-V) fusion virus compared to WT HAdV-C5 (Fig. 3D). We further corroborated the similarity of our reporter viruses to WT HAdV-C5 by examining protein expression of the late adenovirus genes hexon, penton, pV, protein VI, and protein VII during WT, AdNTV, or AdNV infection at 12, 24, and 36 hpi (Fig. 3E). The AdNV reporter showed lower expression of late protein at 24 hpi compared to WT, but both AdNV and AdNTV achieved comparable protein expression to WT HAdV-C5 at 36 hpi, with the exception of a reduction in pVII expression (Fig. 3E). Additionally, fusion of NLuc to pV resulted in the detection of pV at a higher molecular weight in AdNV infection compared to WT.

The AdCTV and AdGTV (Adenovirus mCherry-2A-V and Adenovirus EGFP-2A-V) fluorescent reporters for detecting and visualizing late gene expression showed strong fluorescent signal starting at 24 hpi through to 72 hpi, which was absent at the early 12 hpi timepoint (Fig. 3F). The kinetics of fluorescent protein expression for the late adenovirus genes were consistent with WT HAdV-C5 expression levels (Fig. 3G).

### Specialized viral reporters provide visual detection of viral replication compartments and the early to late phase transition

**Fig 4:**
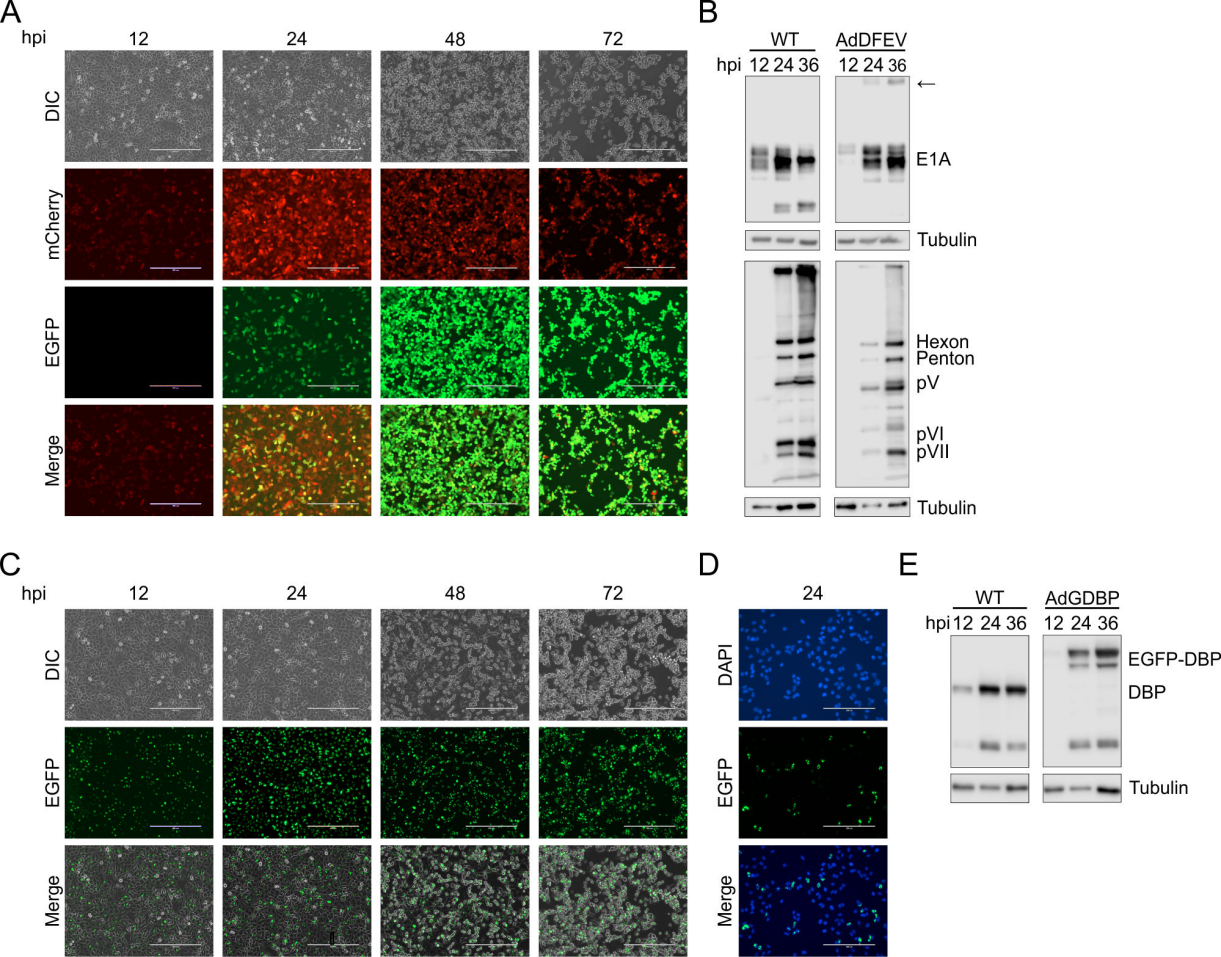
Specialized reporters for dual viral gene expression and viral replication centers recapitulate WT HAdV protein expression patterns. (**A**) Fluorescent microscopy of A549 cells infected with AdDFEV at 12, 24, 48, and 72 hpi. The mCherry signal representing E1A expression is shown in red, EGFP representing pV expression is shown in green, along with a merged image in the bottom panels. (**B**) Western blot for E1A (clone M73) and anti-Ad5 from AdDFEV-infected A549 cells compared to WT HAdV-C5 infected cells at 12, 24, and 36 hpi. Smaller E1A isoforms were undetected in AdDFEV-infected cells. The arrow indicates residual uncleaved reporter-E1A proteins. Tubulin-loading controls are shown. The late viral protein blot for WT-infected cells is the same as shown in Fig. 3E and shown again for comparison. (**C**) Fluorescent microscopy of A549 cells infected with the AdGDBP replication center reporter at 12, 24, 48, and 72 hpi. EGFP signal representing DBP expression and localization is shown in green. (**D**) Fluorescent microscopy of A549 cells infected with the AdGDBP reporter virus at 24 hpi. EGFP signal for DBP expression is shown in green and DAPI stain for cell nuclei is shown in blue. (**E**) Western blot for DBP (clone B6-8) in AdGDBP infected cells compared to WT HAdV-C5 infection for DBP isoforms expression. Scale bars represent (**A and C**) 400 µm and (**D**) 200 µm.

We constructed two unique visual reporter viruses to understand and study more specialized aspects of HAdV replication. The AdDFEV (Adenovirus dual fluor E1A and V; mCherry-2A-E1A and EGFP-2A-V) reporter was designed to report distinct visual fluorescent signals for both early phase and late phase viral gene expressions in the same infected cell. We also constructed the AdGDBP (Adenovirus EGFP-DBP) fusion reporter to mark viral replication compartments (VRCs). Microscopy analysis of AdDFEV-infected A549 cells revealed the red fluorescent E1A signal appearing at 12 hpi, peaking at 24 hpi, and dissipating from 48 to 72 hpi infection (Fig. 4A). Conversely, the green pV signal appeared later at 24 hpi, as expected for a late gene reporter, and intensified up to the 72 hpi timepoint. Merging the images taken at 24 hpi, combining peak red E1A signal and with the incoming green pV signal, revealed overlapping fluorescent signal in a subset of dual-fluor “yellow” cells, indicating the transition between viral early and late phases (Fig. 4A). To ensure AdDFEV expressed appropriate levels of the viral proteins of interest, we infected cells and measured E1A and late viral proteins at 12, 24, and 36 hpi (Fig. 4B). E1A proteins were expressed early and peaked at 24 hpi, whereas late protein expression was highest at 36 hpi for WT virus. Kinetics were slightly delayed for AdDFEV but followed a similar increase in protein expression compared to WT (Fig. 4B). We also detected a faint high molecular weight band for E1A, likely representing residual uncleaved mCherry-E1A, though the majority of the E1A proteins seem efficiently cleaved.

The second specialized HAdV reporter, AdGDBP, uses EGFP fused to DBP to visualize VRCs (34). EGFP signal appeared as discrete puncta at 12 hpi, became more intense at 24 hpi, and maintained a strong signal up to 72 hpi (Fig. 4C). As expected, the EGFP signal appeared strictly nuclear, as confirmed by the exclusive overlap with DAPI stain (Fig. 4D). Protein expression of DBP mirrored that of WT HAdV-C5, but with a shift in the band size, reflecting fusion with the EGFP protein (Fig. 4E).

### Quantification of viral attachment and entry using AdNV

**Fig 5:**
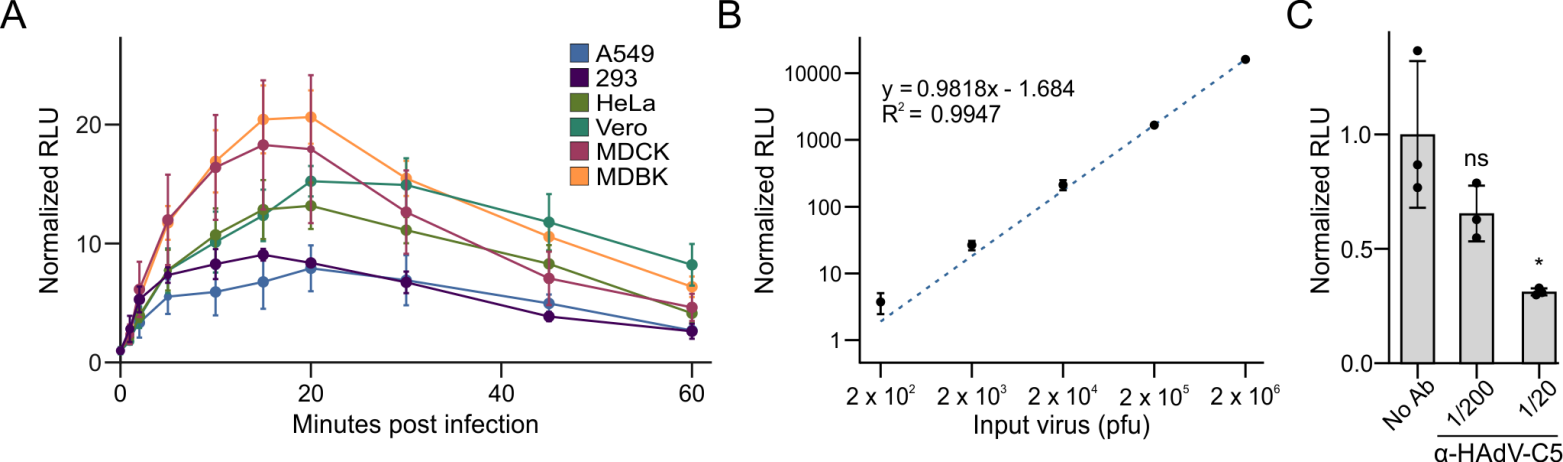
The AdNV bioluminescent virion reporter measures virion attachment and antibody-mediated blockade. (**A**) Attachment time course of the AdNV reporter virus in A549, 293, HeLa, Vero, MDCK, or MDBK cells. Data are normalized to mock control. (**B**) Attachment assay on A549 cells inoculated with increasing amounts of AdNV. Data are normalized to 20 pfu input virus. (**C**) AdNV attachment to A549 cells was disrupted by increasing concentration of an anti-Ad5 antibody. AdNV was pre-incubated with a 1/200 or 1/20 dilution of an anti-Ad5 antibody or control before inoculation on A549 cells at 4°C. Data are normalized to the no antibody control. Data are shown as the grand mean of three biological replicates ± standard error of the mean. Significance was tested with a student’s *t*-test (*<0.05, **<0.01, ***<0.001).

Our final specialized reporter for studying HAdV uses NLuc fused to pV without a self-cleaving 2A sequence. pV is a core component of adenovirus particles; therefore, the virion itself should contain NLuc-V fusion proteins, producing bioluminescent particles that allow for quantification of virus attachment and entry without the need for *de novo* viral gene expression. To test whether AdNV can directly quantify attachment, a variety of cell lines were inoculated with AdNV reporter virus, and NLuc activity was measured over the course of 60 min (Fig. 5A). Importantly, unattached virions were removed via washing before the detection of bioluminescence. Virion attachment increased rapidly, peaking at 20 min postinfection in all cell lines tested (Fig. 5A). These observations are consistent with previous reports of HAdV entry kinetics (35, 36). To examine whether AdNV reporter viruses quantitatively measure binding, we incubated A549 cells with increasing amounts of input virus on ice and measured bioluminescence at 1 hpi. Luciferase activity from AdNV was linearly proportional to the amount of input virus across 5 log units (Fig. 5B). Furthermore, we demonstrated the utility of AdNV to study attachment through a microneutralization assay (Fig. 5C). Before infection of A549 cells, AdNV virus was pre-incubated with either 1/200 or 1/20 dilution of an anti-HAdV-C5 antibody for 15 min. Antibody pre-treatment decreased luciferase activity, reflecting an antibody-mediated blockade to virion attachment (Fig. 5C). The AdNV reporter virus allows for rapid and highly quantitative measures of the earliest stages of infection.

## DISCUSSION

### Adenovirus reporters: a powerful tool to study the viral replication cycle

Replication competent reporter viruses producing bioluminescent or fluorescent signals, have been constructed for a variety of viruses and proven immensely helpful for high-throughput screening, monitoring of viral replication dynamics *in vitro* and *in vivo*, and studying basic or clinical virology (37–42). Adenovirus reporters have also been generated and shown to demonstrate strong utility (43–49). In this work, we expand the adenovirus reporter toolbox with a suite of nine additional replication competent viruses engineered in a WT HAdV-C5 genomic background to investigate immediate early gene expression (AdNTE, AdGTE, and AdCTE), late gene expression (AdNTV, AdGTV, and AdCTV), virion attachment (AdNV), VRC visualization (AdGDBP), and the temporal dynamics between early and late infection (AdDFEV). Notably, all reporter viruses were derived using HAdV-C5, minimizing complications derived from heterogenous genetic backgrounds. This also allows direct comparisons between the different reporter viruses and the isogenic WT virus. In particular, existing reporter viruses contain variable deletions in the E3 region, which although dispensable for growth in culture, adds potentially confounding variables. Moreover, reporter viruses were rescued with the AdenoBuilder platform, enabling facile and rapid production of the current viruses and future variants as needed (50).

### Representative adenovirus genes selected for reporter virus development

E1A was selected as a proxy for immediate early viral gene expression, because it is the first gene transcribed during viral infection (51). Similarly, pV was an ideal candidate to reflect late gene expression, because it is regulated by the major late promoter and is robustly expressed upon the onset of viral genome replication (33, 52–54). As expected, AdNTE and AdNTV recapitulate expression of early and late genes, respectively, allowing detailed analysis of the temporal kinetics of early and late infection.

pV was also selected for developing a virus to study viral attachment and entry. Approximately, 150 copies of pV are packaged into the viral capsid, making it ideal for direct quantification of these specific events (55). Additionally, pV has been successfully expressed as an mCherry fusion without compromising virion integrity (48). Lastly, we chose the E2A-encoded DBP for development of the fluorescence VRC reporter virus, as DBP is expressed during the early phase of infection, is regulated by E1A (56), and is commonly used to demark VRCs (57).

### Reporter design considerations

When selecting reporter elements, we focused on broad utility, high-throughput capability, and non-invasive measurement via rapid and easy enzymatic assays or fluorescence microscopy. NLuc was selected as the enzymatic reporter, because it is roughly 100 times more sensitive than the commonly used firefly luciferase (58). Additionally, its small size of 19 kDa, compared to the 61 kDa size of firefly luciferase, minimizes genomic packaging limitations (59) and potential steric constraints of virion assembly. For visual detection, we selected EGFP and mCherry, because they can be readily detected using common microscopy equipment, have minimal spectral overlap, and use less coding capacity than larger fluors like dTomato (60).

To minimize potential attenuation of E1A or protein V function during infection, the reporter elements were uncoupled from the viral protein by the self-cleaving 2A peptide from porcine teschovirus (38). In contrast to a recent report using an E1A-2A-GFP reporter virus (45), the self-cleaving peptide was highly effective in our hands, with only a small proportion of uncleaved E1A or pV detected. Differences in cleavage efficiency may reflect the location of the reporter element and 2A sequence on the N-terminus or C-terminus.

One advantage of our reporter viruses is the incorporation of a PEST degron fused to the fluorescent reporter elements. PEST degrons are short sequences enriched for proline, glutamate, serine, threonine, and aspartate residues, which act as a protein degradation signal characteristically found in highly regulated cellular proteins (61). Addition of this destabilization signal to GFP reduced protein-half life from ~26 to under 6 h (62, 63). Incorporation of the PEST domain in our adenovirus reporters was done to increase the response ratio by preventing accumulation of reporter signal, which may not accurately reflect current gene expression levels. This should yield reporters that are more responsive to changes in mRNA expression, enabling improved temporal resolution of viral gene expression.

### Potential applications

#### Applications in high-throughput screening

We observed a strong positive correlation between AdNTE virus input and AdNTE bioluminescent output (Fig. 2C). Moreover, treatment with the RNAPII transcription inhibitor ActD abolished the reporting function of AdNTE (Fig. 2B). Taken together, these findings indicate that AdNTE reliably reports early viral gene transcription (Fig. 2A). Likewise, ActD treatment of infected cells demonstrated that late viral gene transcription was required for the reporter function of AdNTV (Fig. 3B). These findings suggest that AdNTE and AdNTV can serve as powerful tools for large-scale drug screening. Indeed, NLuc is ideally suited to high-throughput applications (64). Use of AdNTE or AdNTV would enable the identification of compounds that alter the adenovirus replication cycle. Further, using both reporters would allow one to tease out non-redundant inhibitors that specifically affect the late or early phases of infection (47). Similarly, the mCherry or EGFP fluorescent reporter viruses could be used to quickly and easily visualize changes in the number of infected cells treated with therapeutics for infections done at low MOIs (43).

#### The AdNTE replication assay: a streamlined alternative to the plaque assay

Plaque assays are the gold-standard for quantifying adenovirus titer. This technique typically involves infecting HEK293 cells with serial dilutions of supernatants containing viral progeny then waiting 7 or more days for plaque development. As a high-throughput alternative to the plaque assay, we demonstrated there was no difference between titers determined by plaque assay and luciferase assay (Fig. 2D). Importantly, AdNTE supernatants can be assayed for bioluminescence only 6 h after addition to cells, avoiding the 7 days or more delay typical of plaque assays. Furthermore, because the NLuc assay has >4 log units dynamic range, titration using luciferase assays requires significantly fewer dilutions, if any, compared to the plaque assay. Thus, AdNTE presents a streamlined approach to measuring relative viral replication in experiments with multiple conditions and timepoints as compared to the traditional plaque assay.

#### A tool to study the transition between early and late stages of infection

Investigating the kinetics of the adenovirus replication cycle requires temporal isolation of the early phase, late phase, and transition between the two phases. However, infected cells often exist as a heterogenous population with respect to replication kinetics, regardless of attempts to synchronize infection or cell cycle status. These details make it particularly difficult to discern temporal events at a single cell level. In this regard, we generated AdDFEV, a dual fluor reporter virus that expresses both mCherry-2A-E1A and EGFP-2A-V. Detection of early and late time points coincided with mCherry and EGFP expression, respectively (Fig. 4A). Moreover, the transition between early and late phases was easily detected through overlapping mCherry and EGFP signal, and this was reflected by concurrent E1A and pV protein expression (Fig. 4B). As a dual reporter, this virus effectively allows one to monitor the entire duration of viral replication at the single cell level. These attributes make AdDFEV an ideal candidate for studying viral replication kinetics using existing technologies such as fluorescence-activated cell sorting (FACS), where infected cells can be isolated based on their temporal expression of mCherry and/or EGFP to indicate the early phase, late phase, and the transition between the two (65). Downstream biochemical or molecular applications could then be applied to these enriched cell populations, providing additional insight into the temporal dynamics during infection and the switch between the early and late phase of infection.

#### Visualization of VRC

The AdGDBP reporter virus allows the direct visualization of subnuclear VRCs, which are major sites of viral replication, in infected cells without using standard immunostaining methods (66, 67). DBP morphology changes over the course of infection, appearing as small foci during early time points, and developing into larger, more complex structures at late time points (34, 68). This reporter can be used to visually quantify VRCs, DBP morphology, and possibly aid in identifying small molecules that affect various aspects of DBP function in living cells. For instance, inhibition of the classical nuclear import pathway during infection failed to disrupt DBP nuclear import; however, VRC formation was significantly impaired (69). Overall, this reporter virus could help contribute to our understanding of the spatial and temporal organization of VRCs within the host cell nucleus (68).

#### Rapid assessment of viral attachment

While the viral attachment process for HAdV-C5 is well characterized, the use of the HAdV-C5 vector in gene therapy clinical trials has stimulated further interest regarding virus-cell and virus-immune system interactions (70). Some current approaches to studying viral attachment and entry include using replication incompetent, fluorescent E1-deleted HAdV, infecting with Alexa-fluor labeled HAdV followed by fixing cells and staining with antibody, or using chemically modified capsids (71–74). As a high throughput alternative, we developed the AdNV reporter virus for further exploration of HAdV-C5 attachment.

Using this reporter virus, we detected adenovirus attachment in multiple cell lines over 60 min. Virion uptake and endosomal escape occur within the first 20–30 min of infection, which likely explains the observed peak in AdNV activity by 20 min postinfection (36). A possible explanation for the subsequent decrease in AdNV activity may be related to pV ubiquitination (75). While capsids are docked at the nuclear pore, pV is ubiquitinated, and this is necessary for the proper release of viral genomes into the nucleus. The outcome of pV ubiquitination is unclear; however, the decrease in NLuc activity within this time frame may provide evidence of subsequent pV degradation.

The AdNV reporter virus could represent a rapid system for testing engineered antiviral antibodies or other therapeutics that target viral attachment and entry (76), because this technique can be completed in under an hour and does not require the virus to replicate. In addition, this reporter virus offers a streamlined approach to microneutralization assays, which could also benefit studies of HAdV-C5 with the host immune system (43, 70).

### Future directions and considerations

In this work, we developed a versatile toolbox of nine HAdV-C5 reporter viruses and validated their functions in cell culture. The utility of these reporter viruses could also be extended for use in 3D cell culture, organoids, live cell imaging, and animal models (37, 48, 74, 77–79). Although we have done substantial initial validation, caution and careful optimization must be applied when using these reporter viruses in other infection models, assays, and methodologies. Further, this toolbox provides a conceptual framework for the development of new reporter viruses representing other clinically relevant HAdV species or for investigating distinct events in the viral life cycle (5, 44). This highlights the need for the development of AdenoBuilder type systems for clinically relevant adenoviruses beyond HAdV-C5 (50). In summary, these reporter viruses serve as a powerful new tool to probe discrete stages of the adenovirus replication cycle and provide a streamlined approach to some commonly used techniques and approaches.

## MATERIALS AND METHODS

### Cells, antibodies, and reagents

Human A549 lung cells (ATCC CCL-185), human 293 embryonic kidney cells (ATCC CRL-1573), human HeLa cervical cancer cells (ATCC CCL-2), canine MDCK cells (ATCC CCL-34), bovine MDBK cells (ATCC CCL-22), and African green monkey Vero kidney cells (ATCC CCL-81) were grown in DMEM supplemented with 10% fetal bovine serum at 37°C and 5% CO_2_. Cells were routinely monitored for mycoplasma contamination with MycoAlert (Lonza).

Primary antibodies were mouse anti-tubulin clone 1E4C11 (ProteinTech 66031–1-Ig), mouse M73 anti-E1A (in-house [80]), mouse B6-8 anti-DBP (in-house [81]), and rabbit anti-Ad5 (abcam ab6982). Secondary antibodies were goat anti-rabbit-HRP (Sigma A0545) or goat anti-mouse-HRP (Sigma A4416).

Gibson assembly was performed with HiFi DNA Assembly Master Mix (NEB E2621L). Nanoluciferase activity was quantified with the NanoGlo Luciferase Assay (Promega N1120). Stocks of ActD (Sigma A1410-2mg) were dissolved in DMSO. BstBI was acquired from NEB (R0519L). Transfections were performed with JetPrime (Polyplus 101000001).

### Plasmids

AdenoBuilder plasmids pAd5-B1 to -B7, used to produce recombinant HAdV 5, were a kind gift from F. Bunz (50). Sequences and diagrams for all plasmids generated here can be found in Fig. 1A and Supplemental Files S1–S8.

E1A fusions were created in pAd5-B1. The vector, the reporter gene, and the porcine teschovirus 2A DNA sequence-encoding ATNFSLLKQAGDVEENPGP were amplified by PCR. The three fragments were used for Gibson assembly to insert the reporter gene into the plasmid at the native *E1A* start codon, followed by the in-frame 2A sequence and then the native E1A sequence. Fluorescent reporters were created similarly via a 4-segment Gibson assembly that added the PEST degradation sequence from mouse ornithine decarboxylase upstream of the 2A sequence (82). Nanoluciferase was templated by pNL1.1 (Promega), EGFP by peGFP-C1 (Clontech), mCherry by pmCherry-C1 (Clonetech), and PEST from pNL1.2 (Promega). Final plasmids include pAd5-B1-NLuc-2A-E1A, pAd5-GFP-PEST-2A-E1A, and pAd5-mCherry-PEST-2A-E1A.

pV fusions were created in pAd5-B4 following the same strategy as above. The vector and the reporter genes were amplified by PCR. The pAd5-B1 reporter constructs above were used as templates to amplify reporters with 2A or PEST sequences. Reporters were inserted at the native start codon for pV. Final plasmids include pAd5-B4-NLuc-V, pAd5-NLuc-2A-V, pAd5-GFP-PEST-2A-V, pAd5-mCherry-PEST-2A-V.

The GFP-DBP fusion was created in pAd5-B5. EGFP and the vector were amplified by PCR and assembled with Gibson assembly. GFP was inserted at the native *E2A(DBP*) start codon to create pAd5-B5-EGFP-DBP.

All reporter constructs were verified by sequencing. The following primer pairs were used for gene-specific PCR to confirm the genotype of recombinant viruses. AdDFEV was sequenced using both the AdCTE primers and AdGTV primer pairs.

AdNTE (618 bp product):

NLuc-E1A-F ACACCGGGACTGAAAATGGTCTTCACACTCGAAGA

2A-E1A-R GATAATATGTCTCATTGCGGCCGCAGGTC

AdGTE & AdCTE (933 and 942 bp products, respectively):

GFP/mCherry-E1A-F ACACCGGGACTGAAAATGGTGAGCAAGGGCGA

2A-E1A-R GATAATATGTCTCATTGCGGCCGCAGGTC

AdNV (543 bp product):

Nluc-V-F GGACATTGCGGCCGCCGCCAGAATGCGTTCGC

Nluc-V-R GCGCGCAACGAAGCTATGGTCTTCACACTCGAAGAT

AdNTV (618 bp product):

2A-V-F TTTGCGCTTGGACATTGCGGCCGCAGGTC

Nluc-V-R GCGCGCAACGAAGCTATGGTCTTCACACTCGAAGAT

AdGTV & AdCTV (942 and 933 bp products, respectively):

2A-V-F TTTGCGCTTGGACATTGCGGCCGCAGGTC

GFP/mCherry-V-R GCGCGCAACGAAGCTATGGTGAGCAAGGGCGA

AdGDBP (747 bp product):

GFP-DBP-F GGCCATTGCGGCCGCCTTGTACAGCTCGTCCATG

GFP-DBP-R CTATAGGAGAAGGAAATGGTGAGCAAGGGCGA

### Virus production, replication, and reporter assays

Recombinant adenovirus genomes, along with a WT control, were reconstructed from the pAd5 plasmids by Gibson assembly as described (27, 50). In brief, 125 fmol of each of the seven plasmids were diluted together and digested with BstBI for at least 60 m at 50°C. WT plasmids were substituted with the appropriate reporter constructs to create the desired virus. An equal volume of HiFi DNA Assembly Master Mix was then added, and the reaction was mixed and incubated at 45°C for 1 h. The reaction product was diluted with TE and extracted with an equal volume of phenol/chloroform/isoamyl alcohol (50:49:1). The aqueous phase was recovered and DNA was ethanol precipitated in the presence of glycogen and resuspended in water. A total of 293 cells were then transfected with the purified DNA using JetPrime at a JetPrime:DNA ratio of 2:1 (v/w). Cells and media were recovered ~4.5 days post-transfection and subjected to three rounds of freeze-thaw in a dry ice/ethanol bath. Samples were clarified by centrifugation and used for plaque purification on A549 cells with an agarose overlay.

Viral stocks were prepared by amplifying plaque-purified virus on HEK293 cells. Cells and media were recovered and subjected to three rounds of freeze-thaw in a dry ice/ethanol bath. Samples were clarified by centrifugation and titrated by plaque assay on A549 cells using an Avicel overlay.

Viral replication growth curves were performed by inoculating A549 cells at an MOI of 5. Independent biological replicates were performed for each virus. Cells and supernatant were harvested at the indicated time, subjected to three rounds of freeze-thaw in a dry ice/ethanol bath and titrated by plaque assay on A549 cells.

Nanoluciferase activity was measured as a proxy for viral gene expression, viral replication, and viral yield. A549 cells were inoculated with a nanoluciferase reporter virus at the indicated MOI. The inoculum was removed after 1 h, and cells were washed to eliminate residual nanoluciferase present in the viral stocks. For 0 hpi infections, virus was added to cells, removed immediately, and washed with PBS. In some experiments, ActD was then added to the media at 5 ug/mL. Infection was allowed to proceed up to 24 h to measure gene expression, 24–72 h to measure viral replication, and 12–72 h followed by re-infection with viral supernatants for 6 h to measure viral titers. Nanoluciferase activity was measured by lysing cells in 50 mM Tris pH 7.4, 150 mM NaCl and 0.5% NP40 and analyzing an aliquot with the NanoGlo luciferase assay following the manufacturer’s instructions.

The nanoluciferase attachment assays described below were performed with the AdNV reporter viruses. For attachment assay time course experiments, cell lines were inoculated at an MOI of 5, incubated at 37°C for the indicated time, the inoculum was removed, and cells were washed with PBS. For titration experiments, A549 cells were inoculated in a 96-well plate with a dilution series of virus and incubated at 4°C for 1 h. Virus was removed, cells were washed with PBS, pre-warmed media was added, and cells were incubated for 1 h at 37°C. For antibody blocking experiments, 10,000 pfu of virus was pre-incubated with anti-Ad5 antibody dilutions (or control) and then applied to A549 cells for 15 m at 4°C. The inoculum was removed and cells were washed with PBS. For all assays, cells were then lysed, and luciferase activity was measured as above.

Fluorescent reporter viruses were used to infect A549 cells at an MOI of 5 and imaged using an EVOS FL Auto (Thermo Fisher).

### RT-qPCR

RNA was extracted from infected cells using Trizol (Invitrogen). Reverse transcription was performed with MMLV RT (in-house) using an oligo(dT) primer. qPCR was performed with iTaq Universal SYBR Green Supermix (Bio-Rad 1725122) on a StepOnePlus (Thermo Fisher). Fold changed was calculated by the ∆∆Ct method normalizing to an actin control. The following primers were used:

E1A (13S-10S): F- ACACCTCCTGAGATACACCC; R- TTGCCCAGGCTCGTTAAGC

E1B (E1B-55K): F- GACAATTACAGAGGATGGGC; R- CACTCAGGACGGTGTCTGG

E2A (DBP): F- GGAGCAGCGCGAAACCACCCCC; R- CTCGATACGCCGCCTCATCCGC

E3 (E3-12.5K): F- GAGGCAGAGCAACTGCGCC; R- GCTCTCCCTGGGCGGTAAGCCGG

E4 (E4orf1): F- GGCTGCCGCTGTGGAAGCGC; R- GCCCCCATAGGAGGTATAAC

Actin: F-AGAGCTACGAGCTGCCTGAC; R-AGCACTGTGTTGGCGTACAG

### Western blotting

A549 cells were inoculated at an MOI of 5 with the indicated viruses. Cells were harvested at 12, 24, and 36 hpi and lysed in 50 mM Tris pH 7.4, 150 mM NaCl, and 0.5% NP40. Clarified samples were denatured and separated by SDS-PAGE and transferred to polyvinylidene fluoride membranes. Membranes were blocked with 5% non-fat milk and probed with primary antibodies (E1A M73 clone at 1:10 dilution, DBP B6-8 clone at 1:50 dilution, or anti-Ad5 at 1:10 000 dilution). Antigens were detected by chemiluminescence with either anti-rabbit-HRP or anti-mouse-HRP using an Odyssey Fc Imager equipped with Image Studio (LI-COR).

### Statistics

Data are presented as a mean of three technical replicates ± standard deviation, or the grand mean of three independent biological replications ± standard error. Statistical analysis was performed with Prism (Graphpad), and significance was tested using Student’s *t*-test for pairwise comparisons, or an ANOVA with Dunnett’s *post hoc* analysis to correct for multiple comparisons.

## Data Availability

All data are presented in the accompanying figures. Sequence data for engineered human Ad5 AdenoBuilder plasmids are in the supplemental material.

## SUPPLEMENTAL MATERIAL

File S1 jvi.00207-24-s0001.txt Plasmid sequence for AdGTE.

File S2 jvi.00207-24-s0002.txt Plasmid sequence for AdCTE and AdDFEV.

File S3 jvi.00207-24-s0003.txt Plasmid sequence for AdNTE.

File S4 jvi.00207-24-s0004.txt Plasmid sequence for AdGTV and AdDFEV.

File S5 jvi.00207-24-s0005.txt Plasmid sequence for AdCTV.

File S6 jvi.00207-24-s0006.txt Plasmid sequence for AdNTV.

File S7 jvi.00207-24-s0007.txt Plasmid sequence for AdNV.

File S8 jvi.00207-24-s0008.txt Plasmid sequence for AdGDBP.

ASM does not own the copyrights to Supplemental Material that may be linked to, or accessed through, an article. The authors have granted ASM a non-exclusive, world-wide license to publish the Supplemental Material files. Please contact the corresponding author directly for reuse.
